# Betalains from *Opuntia stricta* peels: UPLC-MS/MS metabolites profiling, computational investigation, and potential applicability as a raw meat colorant

**DOI:** 10.1016/j.heliyon.2024.e39784

**Published:** 2024-10-24

**Authors:** Moufida Chaari, Sarra Akermi, Khaoula Elhadef, Monia Ennouri, Lobna Jlaiel, Mohamed Ali Mosrati, Lotfi Mellouli, Walid Elfalleh, Theodoros Varzakas, Slim Smaoui

**Affiliations:** a Laboratory of Microbial and Enzymes Biotechnology and Biomolecules (LMEBB), Centre of Biotechnology of Sfax (CBS), University of Sfax-Tunisia, Road of Sidi Mansour Km 6, P. O. Box 1177, 3018, Sfax, Tunisia; b Olive Tree Institute, LR16IO01, Sfax, University of Sfax, Tunisia; c Higher Institute of Applied Science and Technology of Mahdia, University of Monastir, Tunisia; d Unity of Analysis, CBS, Sfax, Tunisia; e Laboratory of Molecular and Cellular Screening Processes, Center of Biotechnology of Sfax, University of Sfax, Sidi Mansour Road Km 6, BP “1177”, 3018, Sfax, Tunisia; f Department of Life Sciences, Al Imam Mohamed Ibn Saud Islamic University (IMSIU), 11623, Riyadh, Saudi Arabia; g Higher Institute of Applied Sciences and Technology of Gabes (ISSATGb), University of Gabes, Gabes, 6072, Tunisia; h Department of Food Science and Technology, University of the Peloponnese, Antikalamos, 24100, Kalamata, Greece

**Keywords:** *Opuntia stricta*, Betalains, Molecular docking, Anti-*Listeria monocytogenes*, Raw beef meat quality, Chemometrics

## Abstract

Given consumers', environmental and sustainability apprehensions, the meat industry investigated the natural colorant resources. As proof, betalain, *Opuntia stricta* peels (OSP) pigment, is premeditated in the meat industry. Here, OSP betalains were qualitatively profiled using UPLC-MS/MS, and 7 metabolites were identified: 6 betacyanins and a betaxanthin (arginine-betaxanthin). Molecular docking simulations of cyclo-Dopa-5-O-β-glucoside, as the core betacyanins structure, and the arginine-betaxanthin, displayed the lowest free energies of binding at −8.1 and −7.6 kcal/mol, respectively. These compounds inhibit the *L. monocytogenes* replication and transcription processes by targeting dihydrofolate reductase (DHFR). Then, OSP extracts (0.003, 0.006 and 0.012 %) were incorporated in the raw refrigerated beef meat, and compared to Allura red E129 at 0.002 % for 14 days. By the end of storage, OSP at 0.012 % decreased the chemical oxidation, enhanced the sensory traits, and improved the instrumental color. In addition, chemometrics could distinguish between all samples linking oxidative and microbiological variables to sensory/instrumental color attributes.

## 1 Introduction

In the meat sector, the demand for colorants is notably significant owing to several factors. Firstly, color holds a vital role in influencing consumers' perceptions regarding meat quality and freshness [1]. Vibrant red tones in uncooked meat indicate freshness, captivating consumers and impacting their buying choices. Additionally, colorants contribute to maintaining uniformity across meat products [2]. Consistent coloration guarantees visually appealing products consistently, regardless of variations in meat origins or processing techniques. Regarding this need, the food industries commonly use synthetic red colorants like ponceau 4 R (E124), sunset yellow (E110), carmoisine (E122), Allura red AC (E129), and erythrosine (E127) [3]. The use of these synthetic colorants in meat products is regulated, but their presence highlights the importance of monitoring and understanding their potential effects on health. These health hazards encompass allergic responses, heightened activity levels in children, and even potential connections to specific forms of cancer [4,5]. Hence, there is a need for continued research and development to identify safe and effective natural colorants based on plant extracts that could replace synthetic alternatives.

Many researches have focused on utilizing natural pigments, particularly betalains which are derived from beetroot [6,7], Amaranth [8], and prickly pear [9]. The present study focused on betelains found in purple prickly pear (*Opuntia stricta*) peels (OSP). These pigments showed several valuable effects such as antioxidant, antibacterial, anti-viral and anti-inflammatory properties, indicating promise for alleviating intestinal inflammation [[10], [11], [12]]. These compounds are recognized for their free radical scavenging actions, which contribute to their health-enhancing attributes against prevalent human conditions such as diabetes, hypertension, and hypercholesterolemia [13]. To explore the potential application of OSP extract in meat products, our study focused on its activity against *L. monocytogenes*. This pathogen is particularly important because it is a major cause of foodborne illness, especially in meat. *L. monocytogenes* is particularly targeted due to its ability to survive and grow in refrigerated environments [14]. This bacterium can lead to listeriosis, a severe infection that primarily targets vulnerable persons, including pregnant women, the elderly, newborns, and individuals with weakened immune systems [15]. Hence its high resilience to diverse environmental conditions, makes it a significant threat to public health and a serious problem to socio-economic progress [16]. This is why it became a key focus of our investigation.

On the other side, the use of molecular modeling tools during the last few decades has facilitated the description and understanding of the structural properties and reaction mechanisms of different biological systems, especially the antibacterial [17]. These computational simulation methods focus on the interactions between biomolecules and their possible biological targets by predicting their binding modes, and affinities as well as their inhibitory potential [[18], [19], [20]]. Among the molecular modeling approaches, molecular docking is considered the most widely used method. It illustrates the exact mode of action, reduces experiment costs, and could be applied in various fields [21]. Interestingly, the application of *in silico* tools in food science has rapidly emerged to study the molecular interaction in various food matrices and demonstrate the toxicological effects of foodborne pathogens [22]. Moreover, these innovative techniques are complementary to *in vitro* or *in vivo* assays due to their great ability to simulate different biological processes in an atomic way and provide immediate access to high-resolution molecular data for complex food systems [23].

Several studies have explored meat quality characteristics using a limited number of parameters and manipulations, often without effectively connecting corresponding data. To address the vast array of parameters involved, and to evaluate meat quality, recent approaches have used chemometrics methods *viz*. principal component analysis (PCA) and hierarchical cluster analysis (HCA) coupled with mathematical models [[24], [25], [26]]. The analysis of numerous variables and the integration of all quality parameters are allowed by these methodologies, hence facilitating data dimensionality reduction while preserving the discriminatory power within the data [27,28]. Additionally, they provide a rapid, sample-efficient, and cost-effective approach to food analysis [27,29].

In this study, we aimed to (i) explore the qualitative analyses of betalains compounds of OSP by UPLC-MS/MS, (ii) investigate the molecular docking interactions of the identified compounds by targeting dihydrofolate reductase of *L. monocytogenes*, and (iii) examine the potential of OSP extracts to preserve the raw minced beef meat during refrigerated storage.

## 2 Material and methods

### 2.1 Aqueous *Opuntia stricta* peels extract preparation

Prickly pears (*Opuntia stricta*) were collected from a plot of land located on Afrane Road Sfax, Tunisia (34°47′50.1"N 10°42′01.9"E). Mature fruits without any physical damage were chosen. The peels were cleaned, and dried until a constant weight was reached, utilizing a hot air oven at a temperature set at 40 °C. Following drying, *Opuntia stricta* peels underwent fine grinding using a grinder and subsequent sifting to prepare them for the extraction of betalains. To extract *Opuntia stricta* peels (OSP), the involved extraction conditions were the following: sample-to-solvent ratio of 1:30 (w/v, powder/water at pH 2 acidified with solution of HCL (0.1N), temperature of 40 °C, and extraction duration time of 60 min. Filtration of the extract followed and then centrifugation for 10 min at 10,000×*g* at 4 °C. A rotary evaporator was used to collect and evaporate the supernatants.

### 2.2 Betalains analysis

Spectrophotometric analysis of the concentrations of betacyanins (Bc) and betaxanthins (Bx) was followed at 538 and 480 nm, respectively. The total betalain (Bt) concentration in the OSP extract was determined by these Bc and Bx values, using the method outlined by Righi Pessoa da Silva et al. [30]. Bc and Bx contents were calculated using equations Eq.1, Eq.2), and the results were expressed as mg/g dry extract.

Eq.1 $$Bx=\frac{A\times DF\times MM\left(Bx\right)}{ɛ\times L}$$

Eq.2 $$Bc=\frac{A\times DF\times MM\;\left(Bc\right)}{ɛ\times L}$$

A: Absorbance, DF: Dilution factor, and L: cuvette length. MM (Bc) = 550 g/mol, *ε* = 60,000 L/mol.cm in H_2_O for Bc and MM (Bx) = 308 g/mol, *ε* = 48,000 L/mol.cm in H_2_O for Bx representing the molecular masses (MM) and the molar extinction coefficients (*ε*) used, respectively.

#### 2.2.1 High-resolution UPLC-MS/MS analysis

UPLC-MS/MS (Ultra-performance liquid chromatography-tandem mass spectrometry) was performed using WATER SYNAPT XS high-definition Mass spectrometry, coupled with an Acquity UPLC BEH C18 column (2.1 × 50 mm, 1.7 μm particle size) maintained at 40 °C, at a flow rate of 0.6 mL/min. The injection volume was 1 μL. Using an electrospray Ionization source (source temperature 100 °C, capillary voltage 3 kV, cone voltage 20 V). Argon was used as collision gas (collision energy 4 eV) and nitrogen as desolvation gas (800 L/h). The chromatographic method was launched with 99 % of solvent A (0.1 % formic acid in water (v:v)), followed by a linear gradient from 1 to 40 % solvent B (Acetonitrile) over 12 min and then from 40 % to 100 % B over 2 min. To restore initial conditions, a linear gradient from 100 % B to 100 % A was applied for 3 min. The instrument operated in positive ion mode with a scan range from *m*/*z* 50 to 1200. The mass spectrometer operated with a mass accuracy of 10 ppm.

#### 2.2.2 Antioxidant activity

DPPH (2-diphenyl-1-picrylhydrazyl) and ABTS ((2,2′-azino-bis(3-ethylbenzothiazoline-6-sulfonic acid) assays were used to evaluate the antioxidant capacity of the OSP extract. The DPPH method was determined following the procedure outlined by Xu et al. [31]. In brief, 1 mL of the extract was added to 1 mL of DPPH solution (0.1 mol/L). The absorbance was measured at 517 nm, and the scavenging concentration (IC_50_) was quantified in mg/mL.

The method of Sridhar and Charles was used to determine the ABTS assay [32]. A volume of 500 μL of the extract was added to 1000 μL of ABTS^•+^ solution and subsequently incubated at room temperature for 10 min. The absorbance was then measured at 734 nm. The concentration provides 50 % of radical scavenging activity (IC_50_) and is expressed in mg/mL.

#### 2.2.3 Anti-*Listeria monocytogenes* activity of OSP extract

The minimal inhibitory concentration (MIC) for OSP extract against *L. monocytogenes* ATCC 19117 was determined by the method of Fourati et al. [33]. The concentrations tested ranged from 0.5 to 0.002 mg/mL. Subsequently, each well received 10 μL of a cell suspension containing 10^6^ CFU/mL. Following the incubation period, the wells were exposed to a concentration of 0.5 mg/mL solution of thiazolyl blue tetrazolium bromide (MTT). Further incubation took place at 37 °C for 30 min. Clear wells post-MTT addition indicated the point where microbial growth had stopped.

### 2.3 *In silico* anti-*Listeria monocytogenes* activity of OSP extract

#### 2.3.1 Ligands preparation

The 3D formats of the studied compounds were obtained by drawing their chemical structures using OSRA (Optical Structure Recognition) webserver (https://cactus.nci.nih.gov/cgi-bin/osra/index.cgi) to obtain their SMILES (simplified molecular-input line-entry system) formats. Trimethoprim, an FDA-approved drug was chosen as a positive control and downloaded from Drugbank online (https://go.drugbank.com/). All the SMILES structures were then converted into pdb files using Corina Classic Online demo (https://demos.mn-am.com/corina.html) webserver. The pdb structures were edited using Autodock tools version 1.5.7 by detecting the root to provide flexibility to the chosen ligands to change positions and orientations during molecular docking calculations. The obtained structures were saved as pdbqt files.

#### 2.3.2 Dihydrofolate reductase (DHFR) preparation

The dihydrofolate reductase (DHFR) was employed as a possible antibacterial target against the pathogenic bacterium *L. monocytogenes* ATCC 7644. The FASTA sequence of the chosen protein was obtained from NCBI database (PDB ID: OYN31508). As no crystallographic structure was available, molecular homology simulation using SwissModel webserver (https://swissmodel.expasy.org/) was effectuated to predict similarity and obtain an accurate model. Later, the acquired model was subjected to Profunc webserver (https://www.ebi.ac.uk/thornton-srv/databases/profunc/index.html) to predict the active site amino acids and to analyze the Ramachandran plots using the PROCHECK analysis tool. Additionally, the pdb structure of DHFR was checked and cleaned from water molecules and any other fixed ligands and cofactors using Discovery Studio software version 2017 R2 (Dassault Systemes BIOVIA, 2017) and saved as a pdb format.

#### 2.3.3 Molecular docking calculations

Molecular docking simulations were conducted out using Autodock vina software version 1.5.7 [34] following the methodology previously outlined by Akermi et al. [35]. All the necessary charges such as Kollman and Gasteiger were added to the selected target structure. Hydrogen atoms were then merged, and the protein was saved in pdbqt format. The grid box was built around the binding site using a standard size of 80 x 80 x 80 with a grid spacing of 0.375 Å. The coordinates of the grid center were set based on the residues of the binding site within the proteins with *x=* −0.84, *y=* 0.438 and *z=* −2.618. The exhaustiveness value was set to 100 with many modes of 200 and *energy_range* to 4. Finally, the obtained receptor-ligand complexes were analyzed, ranked based on their free energies of binding scores and visualized using BIOVIA® Discovery Studio® software version 2017 R2 (Dassault Systems BIOVIA, 2017, San Diego, CA, USA).

### 2.4 Preparation of raw minced beef meat

Fresh meat beef, sourced from a local supplier (slaughterhouse of Sfax, Tunisia), was taken to the laboratory under chilling conditions, where hygienic mincing took place using a sterile meat grinder with a 3 mm diameter mincing plate. A total of 6 kg of meat was used (5 treatments × 5 time periods × 3 Triplicate analysis).

**Fig. 1:**
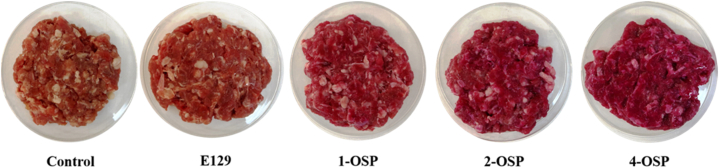
Visual aspects of minced beef meat treated with E129, 1-OSP and 2-OSP, 4-OSP, and non-treated (control) before storage.

The different portions were separately prepared: The initial untreated sample was the control sample, while the second sample acted as a positive control and was treated with 0.002 % Allura Red E129, a synthetic colorant commonly utilized in the food industry (Fig. 1). According to the European Food Safety Authority (EFSA), the maximum permitted level of E129 introduced in meat is 25 mg/kg [36].

In addition, three concentrations of the extracts: 1-OSP (0.003 %), 2-OSP (0.006 %), and 4-OSP (0.012 %), corresponding to 1 × MIC, 2 × MIC, and 4 × MIC of *L. monocytogenes* ATCC 19117, respectively, were applied to the meat sample. Evaluation was done on days 0, 3, 7, 10, and 14 after all meat samples were placed in sterile plastic bags and kept at 4 °C.

#### 2.4.1 Physicochemical analysis of raw minced beef meat

To evaluate protein oxidation, carbonyl content (CC) was measured using the 2,4-dinitrophenylhydrazine (DNPH) method, detailed by Mtibaa et al. [37]. The method is based on the reaction between the DNPH with protein carbonyl compounds to form a 2,4-dinitrophenyl (DNP) hydrazone product which displays a maximum absorbance peak at around 370 nm. One gram of ground beef was homogenized in 10 mL of 0.15 M KCl buffer for 60 s. A 50 μL aliquot of this homogenate was transferred into an Eppendorf vial containing 1 mL of 10 % (w/v) trichloroacetic acid (TCA). After centrifuging the samples for 5 min at 3000×*g*, the supernatant was discarded. Then, 1 mL of HCl (2M) with 0.2 % DNPH was added. The samples were incubated at room temperature for 1 h, with vortexing every 20 min. Following incubation, 1 mL of 10 % TCA was added, vortexed, and samples were centrifuged again for 10 min at 3000×*g*. The supernatant was removed, and washing of the pellets occurred twice using 1.5 mL of ethanol/ethyl acetate (1:1; v/v), followed by shaking and centrifuging for 5 min at 10,000×*g*. After the DNPH residues were completely removed, the pellets were dissolved in 1.5 mL of 20 mM sodium phosphate buffer pH 6.5 (with 6 M guanidine hydrochloride), and centrifuged for 5 min at 4000×*g*. and expressed as nmol carbonyl/mg of protein. The concentration of DNP hydrazones is considered by quantifying reacted DNPH spectrophotometrically based on the absorption of 22,000 M^−^^1^cm^−^^1^ at 370 nm.

Peroxide values (PV), conjugated dienes (CD), and thiobarbituric acid reactive substances (TBARS) were evaluated as indices of lipid oxidation. The quantification of PVs in all meat samples followed the Folch method as explained by Mtibaa et al. [37], and the results were reported as milliequivalents of peroxide per kilogram of meat.

CD values were determined based on the protocol outlined by Mtibaa et al. [37]. The molar extinction coefficient (25,000 M^−1^ cm^−1^) was used to calculate the concentration of CD and results were expressed as micromoles per milligram of meat.

TBARS values were determined according to the method detailed by Eymard et al. [38]. The absorbance of meat samples was assessed spectrophotometrically, and the results were reported as milligrams of malonaldehyde (MDA) per Kg of meat.

- Microbiological analysis

Ten g of each sample (control, E129, 1-OSP, 2-OSP, and 4-OSP), were blended in a stomacher using 90 mL of sterilized peptone water and homogenized. Inoculation of the samples onto solid culture medium plates was performed by decimal dilutions. Plate Count Agar (PCA) at 30 °C for 48 h [39] and at 7 °C for 10 days were employed for the enumeration of Aerobic Plate Counts (APC) and Psychrotrophic Total Counts (PTC), respectively [40].

- Instrumental color assessment

The color assessment of the samples was conducted using a spectrophoto colorimeter, MiniScan XE™ (Hunter Associates Laboratory Inc., Reston, VA, USA), equipped with a 22-mm aperture and a 10° observer, and calibrated with a white tile. These results were calculated based on a 10° standard observer and D65 illuminant. Brightness level, ranging from 0 (black) to 100 (white) is represented by *L*∗ (Lightness) component. The green-red axis shows the *a*∗ (Redness) component, where positive values signify redder hues and negative values correspond to greener shades. The blue-yellow axis depicts the *b*∗ (Yellowness) component, with positive values indicating yellower shades and negative values indicating bluer tones.

- Sensory evaluation

To assess the sensory attributes (odor, color, appearance, and overall acceptability) of all meat samples, a panel of 20 non-trained evaluators (15 females, 5 males) from graduate students and the administrative staff of the University of Sfax (Tunisia) was engaged. The selection criteria for participants included ages between 23 and 46 and non-smoking habits. Each panel member conducted evaluations for the meat samples (Control, treated with E129, and treated with 1-OSP, 2-OSP, and 4-OSP extracts) on different analysis days (0, 3, 7, 10, and 14). Each member received one sample of each treatment randomly numbered and served. The assessments were carried out using a hedonic scale ranging from 1 to 9, representing degrees of dislike to like [41].

### 2.5 Statistical analysis

Measurements were conducted at intervals of 0, 3, 7, 10 and 14 days of storage, with three replications performed at each time point. A one-way analysis of variance (ANOVA) was carried out for all variables. The statistical significance of differences between mean values was considered using triplicate measures and Tukey's test with SPSS (Statistical Package for the Social Sciences, SPSS Ltd. Woking, United Kingdom) software version 26.

Principal component analysis (PCA) and hierarchical cluster analysis (HCA) were the two chemometric approaches used. Each cluster was defined using the Ward approach and the squared Euclidean distance matrix, producing a dendrogram. The XLSTAT program for Windows (v.2014, Addinsoft, New York, USA) was used to accomplish these methods.

## 3 Results and discussion

### 3.1 Quali-quantitative OSP betalains analysis

#### 3.1.1 Quantification of betacyanins and betaxanthins

The concentrations of betacyanins (Bc) and betaxanthines (Bx) in the OSP extract were 2.55 ± 0.01 and 1 ± 0.10 mg/g, respectively, with Bt equal to 3.55 mg/g. As indicated by previous research sources, the total betalain content in the *Cactaceae* species exhibits significant variation. García-Cayuela et al. [42] studied the concentration of Bt in the Spanish Morada variety of *Opuntia ficus*-indica purple peels extract and reported a value of 2.0 mg/g, with a Bc and Bx equal to 1.0 and 0.17 mg/g, respectively. This variability may be attributed to the geographical location where the fruits were cultivated and harvested [43]. Additionally, the variations in betalain concentrations can be influenced by factors such as extraction ratio, time, and temperature [44]. These variables play a crucial role in determining the final content produced, and consequently, the efficiency of betalain extraction.

#### 3.1.2 Metabolite identification using UPLC-MS/MS analysis

**Fig. 2:**
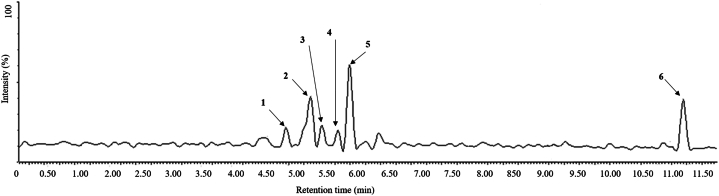
UPLC-MS/MS profile of *Opuntia stricta* aqueous extract, with letters 1–6 corresponding to the mentioned components in Table 1.

The betalains profile of OSP extract was analyzed using UPLC-MS/MS (Fig. 2). Retention times (Rt), UV/Vis spectra, and MS spectral data of the identified compounds are presented in Table 1. The major betalains found belonged to the betacyanin group. Thus, compound 2 was tentatively identified as 15-hydroxybetanidin-5-O-β-glucoside: Rt: 5.36 min and *m/z*: 566.42 (Table 1). This compound was identified following the study of Herbach, Stintzing, and Carle [45] where the compound was reported at *λ*_max_ 526 nm and *m/z* 567. The formation of this compound could result from the incorporation of a water molecule (*m/z* 18) into a neobetanin molecule (*m/z* 549) by non-enzymatic modification under naturally acidic conditions. Additionally, neobetanin (14,15-dehydrobetanin) may result from betanin or isobetanin precursors by dehydrogenase activity [46].

**Table 1:** Molecular formula, retention times (Rt), UV–Vis data and mass spectral results for betalains identified in OSP extract by UPLC-MS/MS.

Peak Number	Compounds	Molecular formula	UV–vis maximum (nm)	Retention time (min)	[M+H]^+^*m*/*z*	MS/MS
Betacyanins						
**1**	Unknown	–	–	4.95	525.29	90.97-117.08-129.05-158.96-185.11-226.95-294.93-301.14-317.14-**359.23**-420.89-430.9-449.36-453.34-475.89-503.31
**2**	15-Hydroxybetanidin 5- O- β –glucoside		529	5.36	566.42	118.08-129.05-226.95-301.14-**359.23**-430.91-449.36-488.86-548.42
**3**	Unknown	–	–	5.55	613.33	549
	Neobetanin (14,15- dehydrobetanin)	C_24_H_24_N_2_O_13_	467	–	–	549
	Betanidin-5-O-(6-O-malonyl)-β-glucoside, bi-decarboxylated	C_24_H_24_N_2_O_13_	467	–	–	549
**4**	Unknown	–	–	5.8	657.36	
**5**	Betanidin 5- O-(6′-O- β malonyl) β -glucoside (phyllocactin)	C_27_H_28_N_2_O_16_	535	5.99	637	118.08-129.05-226.95-301.14-**359.23**-430.91-453.34-497.39-548.4-566.43-567.43-634.88
**5′**	Isobetanidin 5- O-(6-O-malonyl)- β -glucoside (Isophyllocactin)	C_27_H_28_N_2_O_16_	534	–	637	–
	**Cyclo-Dopa-5-O-β-glucoside**	C_15_H_19_NO_9_	283	**-**	–	**359.23**
**Betaxanthins**						
**6**	**Arginine-Betaxanthin**	C_15_H_21_N_5_O_6_	470	11.44	367.06	55.54-96.62-129.05-161.06-205.5-235.20-240.23-241.23-301.14-333.86-351.16

Betanidin 5- O-(6′-O- β malonyl) β –glucoside (phyllocactin) or its isomer Isobetanidin 5- O-(6-O-malonyl)- β -glucoside (Isophyllocactin) were putatively identified by the precursor ion at *m/z* 637 (Rt: 5.99 min), as the compound 5 and 5’, respectively which are typical compounds of the *Cactaceae* family [46]. In fact, Kumorkiewicz-Jamro et al. [47] and Sutor-Świeży et al. [48] mentioned that the formation of dehydrogenated and decarboxylated derivatives is likely to take place in a highly acidic environment, which confirms the presence of neobetanin (14,15-dehydrobetanin) and betanidin-5-O-(6-O-malonyl)-β-glucoside, bi-decarboxylated. In addition, Fernandez-Lopez et al. [49] found that pH significantly influenced both the color and the formation of degradative pigments, indicating that the degradation mechanism of betacyanins in cactus pears is pH-dependent. Additionally, peak 6 was tentatively assigned as Arginine-betaxanthin (Rt = 11.44 min) due to the presence of the precursor ion [M+H]^+^ at *m/z* 367 corresponding to the combination of betalamic acid with arginine (Fig. 2). In fact, genetic diversity within *Opuntia* genotypes plays a pivotal role in shaping the betalain profile, indicating that variations in betalain composition can be attributed to genetic factors within the *Opuntia* species [50].

The identification of Cyclo-DOPA-5-O-β-glucoside was realized using the MS/MS spectra of the compounds 1 (Rt: 4.95 min), 2 (Rt: 5.36 min) and 5 (Rt: 5.99 min). These molecules undergo fragmentation in this process, revealing the same characteristic fragments 359.23. This strongly suggests that they contain the Cyclo-DOPA-5-O-β-glucoside moiety or a closely related structure. In addition, the presence of Cyclo-DOPA-5-O-β-glucoside with an *m/z* of 359.23 can be explained by the ionization and fragmentation processes that occur during mass spectrometry analysis (358 + 1). This fact was confirmed by Herbach et al. [51].

### 3.2 *In vitro* biological activities of OSP extract

The scavenging of free radicals by the OSP extract exhibited notable effectiveness, reflected in an IC_50_ (DPPH) value of 0.9 ± 0.10 mg/mL. While, the IC_50_ of ABTS scavenging value was equal to 0,457 ± 0.014 mg/mL. This might be explained by the differing nature of the active compounds in the extract or the different interactions between the extract's components and each type of radical. This potency aligns with findings from various studies investigating the antioxidant capabilities of prickly pear extracts [9,52]. For instance, research on the hydroethanolic (80 %; v/v) extract of *Opuntia engelmannii* peels revealed an IC_50_ of DPPH value equal to 1.96 mg/mL [53]. In another study, Mannoubi [54] found that the extract of *Opuntia stricta* fruit with 80 % acetone exhibited an IC_50_ (ABTS) value of 1.793 mg/mL.

Hence, the antioxidant potential of OSP extract is attributed not only to the presence of polyphenols but also to the concentration of betalains [55]. Nevertheless, the variability of the antioxidant activity could be influenced by several factors such as postharvest quality, prickly pear varieties, and exposure to elevated temperatures.

The MIC of OSP extract against *L. monocytogenes* was determined at 0.03 mg/mL, indicating a potent antibacterial activity. Higher MIC values against *L. monocytogenes* were obtained by Melgar et al. [53] when *Opuntia ficus*-indica (0.15 mg/mL) and *Opuntia engelmannii* (0.3 mg/mL) peel extracts were used. Therefore, high concentrations of phytochemical components, including betalains, polyphenols, and other bioactive compounds depict the extract's antibacterial effects. These phytochemicals are renowned for their antimicrobial properties and can inhibit bacterial growth [56].

These data hold promise for the development of novel antioxidant and antibacterial agents derived from prickly pear peel extract, which could be utilized in various food and pharmaceutical applications.

### 3.3 *In silico* anti-*Listeria monocytogenes* activity of betalains compounds

Dihydrofolate reductase (DHFR) is an essential enzyme in the bacterial folic acid pathway, playing a key role in reducing dihydrofolate to tetrahydrofolate to facilitate thymidylate biosynthesis [57]. Due to its critical role, DHFR has emerged as a promising target for combating bacterial resistance to conventional antibiotics as its blocking could affect DNA translation, RNA transcription, and protein replication processes, as well as cell proliferation arrest [58]. In the present study, all the compounds of OSP extract, previously detected and identified by UPLC-MS/MS, were subjected to a molecular docking approach to assess their antibacterial potential against the foodborne pathogen *L. monocytogenes* by targeting the DHFR. Molecular homology results depicted that the obtained model has an estimated identity percentage equal to 99.38 %. Additionally, the stereochemical quality evaluation of the model showed a Global Model Quality Estimate (GMQE) value equal to 0.98. Additionally, the most favored region of Ramachandran plots was predicted to be higher than 90 % [59]. In this sense, molecular docking calculations revealed that all the studied betalains compounds of OSP extract had effective antibacterial properties against the tested bacterium by inhibiting DHFR activity as mentioned in Table 2. Based on the docking scores (Gibbs free energy of binding (ΔG)), the compounds cyclo-Dopa-5-O-β-glucoside and Arginine-Betaxanthin displayed the lowest free energies of binding (−8.1 kcal/mol) and (−7.6 kcal/mol), respectively, as compared to the chosen control trimethoprim (−7.4 kcal/mol). It is important to mention that trimethoprim is an antibiotic approved by the Food and Drug Administration (FDA) and prevents bacteria from producing the necessary folic acid for DNA synthesis [60]. A previous study reported that prickly pear extract showed an inhibitory effect on the growth of *S. aureus*, *L. monocytogenes* and *S. typhimurium*. The observed effect was attributed to the source of betacyanins [61]. Another study conducted by Chaari et al. [56] indicated that Bc and Bx compounds could inhibit pathogenic bacteria replication and transcription processes by impeding DNA and RNA polymerase activities. Although its richness in bioactive compounds, *Opuntia* species are still not well valued. Research showing their exact antibacterial mechanism of action against pathogens is still lacking in the literature and not well documented. There is no investigation on molecular docking studies conducted on Betanidin-5-O-(6-O-malonyl)-β-glucoside, Cyclo-Dopa-5-O-β-glucoside, Phyllocactin, Isophyllocactin, 15-Hydroxybetanidin 5- O- β -glucoside, or Arginine-Betaxanthin within the provided contexts.

**Table 2:** Docking scores of *Opuntia stricta* aqueous extract components against DHFR target.

Compounds	Free energy of binding (Kcal/mol)	Number of interacting residues	H-bond interactions
15-Hydroxybetanidin 5- O-b-glucoside	−7.4	–	–

**Cyclo-Dopa-5-O-β-glucoside**^a^	**−8.1**	**6**	**VAL5, ALA7, TRP6, TYR101, SER49 and ASN18**

Neobetanin (14,15- dehydrobetanin)	−7.5	–	–
Betanidin-5-O-(6-O-malonyl)-β-glucoside, bi-decarboxylated	−6.7	–	–
Betanidin 5- O-(6′-O- β malonyl) β -glucoside (phyllocactin)	−7.2	–	–
Isobetanidin 5- O-(6-O-malonyl)- β -glucoside (Isophyllocactin)	−7.0	–	–

**Arginine-Betaxanthin**^a^	**−7.6**	**3**	**VAL5 and ILE14**

Trimethoprim (control)	−7.4	–	–

a Interaction details of Cyclo-Dopa-5-O-β-glucoside and Arginine-Betaxanthin against DHFR *L. monocytogenes* target.

**Fig. 3:**
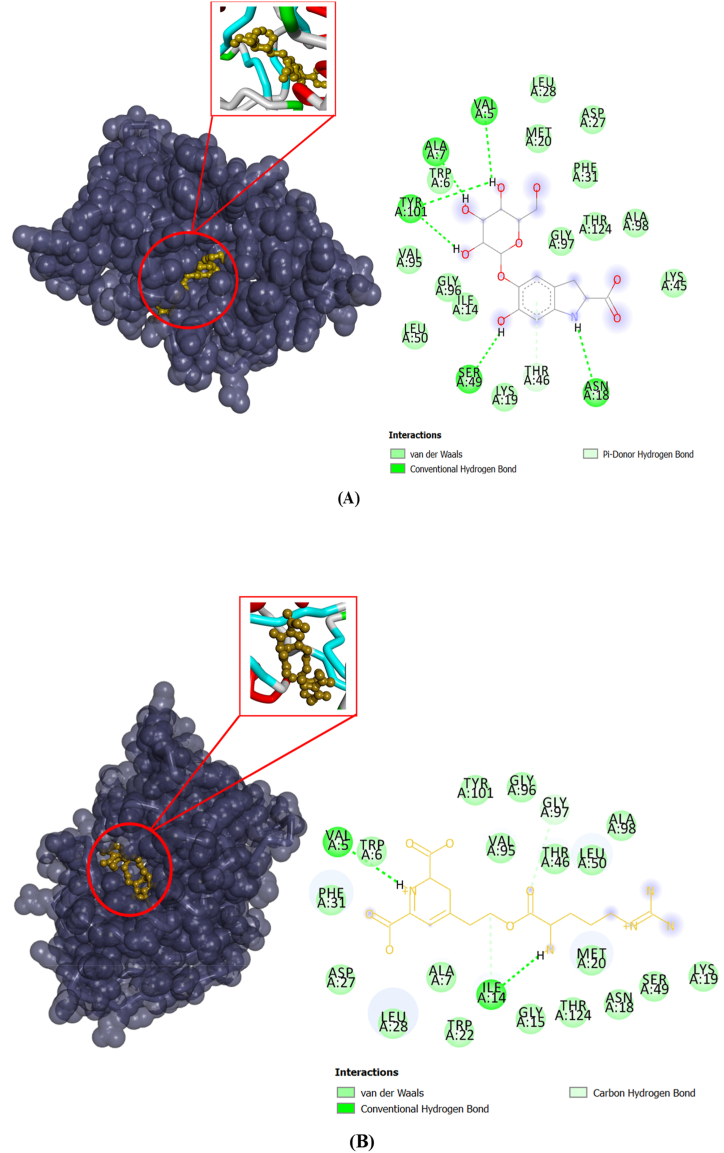
Dihydrofolate reductase (DHFR) of *L. monocytogenes* complexed with Cyclo-Dopa-5-O-β-glucoside (**A**) and Arginine-Betaxanthin (**B**).

Interactions details of the two compounds Cyclo-Dopa-5-O-β-glucoside and Arginine-Betaxanthin that showed the best docking scores and the highest affinities and inhibitory potential against the selected target dihydrofolate reductase of *L. monocytogenes* are summarized by Table 2 and Fig. 3A and B. The complex of Cyclo-Dopa-5-O-β-glucoside against DHFR target displayed the presence of van der Waals interactions with LEU28, MET20, ASP27, PHE31, GLY97, THR124, ALA98, LYS45, THR46, LYS19, ILE14, LEU50, GLY96, VAL95 and conventional hydrogen bonds via ASN18, SER49, TRP101, ALA7 and VAL5 (Fig. 3A).

On the other side, Arginine-Betaxanthin exhibited an inhibitory effect against DHFR by interacting with the selected enzyme via van der Waals interactions with TYR101, GLY96, GLY97, VAL95, THR46, LEU50, ALA98, LYS19, SER49, MET20, ASNA18, THR124, GLY15, TRP22, ALA7, LEU28, ASP27, PHE31, TRP6 and conventional hydrogen bonds with ILE14 and VAL5 (Fig. 3B).

Therefore, OSP extract compounds could be applied as effective antibacterial agents to combat infections caused by foodborne pathogens and could be a potent alternative to tackle bacterial resistance to conventional antibiotics. However, these valuable compounds need deeper research related to their multiple modes of action which may facilitate their valorization in sustainable and environmentally friendly processes.

### 3.4 Analysis of raw minced beef meat samples

#### 3.4.1 Microbiological analysis

The APC in raw minced beef meat was monitored during refrigerated storage for 14 days. As presented in Table 3, the highest APC levels were observed in both non-treated meat samples (control) and those treated with E129. However, the addition of OSP (*Opuntia stricta* peels) extract showed a significant (*P* < 0.05) reduction in the growth rate of APC. By the end of storage, meat samples treated with 1-OSP, 2-OSP and 4-OSP extracts exhibited APC levels of 6.38, 5.98, and 5.32 log CFU/g, respectively. Consequently, the meat samples treated with OSP extracts maintained APC levels below the recommended limit (6.7 log CFU/g) [62]. Furthermore, these outcomes indicated a notable decrease (*P* < 0.05) in APC with a rising concentration of OSP extract. Comparatively, Parafati et al. [63] mentioned that beef burger patties treated with prickly pear extract depicted an APC equal to 6.66 log CFU/g, after 8 days of storage at 4 °C. This provides additional evidence supporting the efficacy of prickly pear extract in retarding bacterial growth in meat products.

**Table 3:** Effect of E129 (0.002 %), 1-OSP (0.003 %), 2-OSP (0.006 %), and 4-OSP (0.012 %) extracts on microbiological, oxidation, instrumental color and sensory parameters of raw minced beef stored at 4 °C for 14 days.

	Days of storage					
	Samples	0	3	7	10	14
**APC (log CFU/g)**	**Control**	2.29 ± 0.029^aA^	4.67 ± 0.10^cB^	5.91 ± 0.20^cC^	6.95 ± 0.14^dD^	7.59 ± 0.12^dE^
	**E129**	2.27 ± 0.031^aA^	4.69 ± 0.11^cB^	5.89 ± 0.18^cC^	6.91 ± 0.17^dD^	7.61 ± 0.14^dE^
	**1-OSP**	2.28 ± 0.03^aA^	4.32 ± 0.09^bB^	4.87 ± 0.17^bC^	5.25 ± 0.10^cD^	6.38 ± 0.11^cE^
	**2-OSP**	2.23 ± 0.028^aA^	3.85 ± 0.12^aB^	4.26 ± 0.13^aC^	4.98 ± 0.09^bD^	5.98 ± 0.13^bE^
	**4-OSP**	2.25 ± 0.032^aA^	3.65 ± 0.04^aB^	4.05 ± 0.10^aC^	4.59 ± 0.11^aD^	5.32 ± 0.10^aE^
**PTC (log CFU/g)**	**Control**	2.12 ± 0.07^aA^	4.20 ± 0.12^cB^	5.18 ± 0.17^cC^	5.95 ± 0.14^dD^	6.59 ± 0.10^cE^
	**E129**	2.06 ± 0.07^aA^	4.24 ± 0.11^cB^	5.21 ± 0.21^cC^	5.87 ± 0.15^dD^	6.61 ± 0.17^cE^
	**1-OSP**	2.08 ± 0.07^aA^	3.72 ± 0.09^bB^	4.19 ± 0.11^bC^	4.31 ± 0.11^cD^	5.78 ± 0.06^bE^
	**2-OSP**	2.09 ± 0.07^aA^	3.58 ± 0.02^abB^	4.06 ± 0.12^bC^	4.33 ± 0.09^bD^	5.51 ± 0.03^aE^
	**4-OSP**	2.11 ± 0.07^aA^	3.33 ± 0.10^aB^	4.00 ± 0.14^aC^	4.18 ± 0.08^aD^	5.05 ± 0.15^aE^
**Carbonyl content (nmol carbonyl/mg of protein)**	**Control**	0.53 ± 0.036^aA^	0.63 ± 0.010^aB^	0.92 ± 0.012^cC^	1.23 ± 0.010^cD^	1.87 ± 0.006^cE^
	**E129**	0.49 ± 0.010^aA^	0.64 ± 0.021^aB^	0.97 ± 0.015^cC^	1.19 ± 0.020^cD^	1.94 ± 0.02^cE^
	**1-OSP**	0.48 ± 0.010^aA^	0.60 ± 0.021^aB^	0.71 ± 0.010^bB^	0.96 ± 0.011^bC^	1.31 ± 003^bD^
	**2-OSP**	0.52 ± 0.015^aA^	0.58 ± 0.010^aA^	0.68 ± 0.007^abB^	0.72 ± 0.015^aB^	0.97 ± 0.01^aC^
	**4-OSP**	0.47 ± 0.010^aA^	0.54 ± 0.015^aA^	0.61 ± 0.015^aB^	0.68 ± 0.014^aB^	0.84 ± 0.005^aC^
**PV (meq of peroxide/Kg)**	**Control**	3.74 ± 0.105^aA^	4.15 ± 0.156^bB^	5.17 ± 0.240^cC^	6.21 ± 0.260^cD^	7.87 ± 0.310^dE^
	**E129**	3.70 ± 0.085^aA^	4.12 ± 0.132^bB^	5.15 ± 0.160^cC^	6.20 ± 0.193^cD^	7.84 ± 0.240^dE^
	**1-OSP**	3.62 ± 0.075^aA^	4.07 ± 0.122^bB^	4.69 ± 0.155^bBC^	5.77 ± 0.180^bC^	7.53 ± 0.226^cD^
	**2-OSP**	3.60 ± 0.040^aA^	4.01 ± 0.055^abB^	4.47 ± 0.095^abB^	5.37 ± 0.110^aC^	6.64 ± 0.182^bD^
	**4-OSP**	3.72 ± 0.015^aA^	3.84 ± 0.045^aA^	4.35 ± 0.091^aB^	5.23 ± 0.111^aC^	6.14 ± 0.157^aD^
**CD (μmol/mg)**	**Control**	0.22 ± 0.031^aA^	0.72 ± 0.035^bB^	0.91 ± 0.041^bC^	0.84 ± 0.046^bcB^	0.81 ± 0.06^bB^
	**E129**	0.25 ± 0.024^aA^	0.74 ± 0.030^bB^	0.97 ± 0.036^bD^	0.86 ± 0.040^cC^	0.84 ± 0.049^bC^
	**1-OSP**	0.21 ± 0.015^aA^	0.51 ± 0.023^abB^	0.61 ± 0.029^aB^	0.59 ± 0.030^abB^	0.51 ± 0.040^aB^
	**2-OSP**	0.24 ± 0.013^aA^	0.36 ± 0.018^aA^	0.57 ± 0.024^aC^	0.49 ± 0.026^aB^	0.47 ± 0.034^aB^
	**4-OSP**	0.21 ± 0.010^aA^	0.31 ± 0.016^aA^	0.51 ± 0.020^aC^	0.43 ± 0.025^aB^	0.41 ± 0.031^aB^
**TBARS (mg MDA-eq/Kg)**	**Control**	0.11 ± 0.002^aA^	0.84 ± 0.003^bB^	1.22 ± 0.006^bC^	2.13 ± 0.007^cD^	3.24 ± 0.009^cE^
	**E129**	0.12 ± 0.00^2aA^	0.88 ± 0.005^bB^	1.13 ± 0.003^bC^	2.05 ± 0.005^cD^	3.15 ± 0.005^cE^
	**1-OSP**	0.11 ± 0.002^aA^	0.68 ± 0.001^abB^	0.72 ± 0.001^aC^	1.41 ± 0.002^bD^	2.12 ± 0.003^bE^
	**2-OSP**	0.10 ± 0.002^aA^	0.52 ± 0.004^aB^	0.61 ± 0.002^aC^	1.29 ± 0.003^abD^	1.85 ± 0.004^aE^
	**4-OSP**	0.11 ± 0.002^aA^	0.48 ± 0.001^aB^	0.53 ± 0.001^aC^	1.14 ± 0.004^aD^	1.73 ± 0.001^aE^
***L∗***	**Control**	50.99 ± 2.55^dC^	50.16 ± 2.50^cC^	47.79 ± 2.38^dBC^	46.30 ± 2.31^cB^	43.89 ± 2.19^cA^
	**E129**	38.63 ± 1.93^cC^	37.71 ± 1.88^bBC^	36.49 ± 1.82^cB^	36.36 ± 1.81^bB^	33.41 ± 1.67^bA^
	**1-OSP**	34.84 ± 1.66^bC^	33.21 ± 1.66^abBC^	32.45 ± 1.62^bB^	32.88 ± 1.60^abB^	31.21 ± 1.56^aA^
	**2-OSP**	33.83 ± 1.65^abB^	33.07 ± 1.65^abB^	32.36 ± 1.61^bAB^	32.25 ± 1.61^abAB^	31.12 ± 1.55^aA^
	**4-OSP**	32.41 ± 1.60^aB^	32.15 ± 1.60^aAB^	31.71 ± 1.58^aAB^	31.43 ± 1.57^aA^	31.01 ± 1.55^aA^
***a∗***	**Control**	12.34 ± 0.49^aD^	11.62 ± 0.46^aCD^	10.35 ± 0.31^aC^	7.48 ± 0.22^aB^	6.25 ± 0.187^aA^
	**E129**	14.32 ± 0.57^abC^	13.29 ± 0.53^bB^	13.17 ± 0.39^bB^	12.15 ± 0.36^bAB^	11.74 ± 0.35^bA^
	**1-OSP**	17.68 ± 0.70^bD^	14.87 ± 0.59^bcC^	13.87 ± 0.41^bBC^	13.23 ± 0.39^bcB^	11.92 ± 0.35^bA^
	**2-OSP**	18.49 ± 0.73^bE^	16.86 ± 0.67^cD^	15.51 ± 0.46^cC^	15.04 ± 0.45^cB^	14.51 ± 0.43^cA^
	**4-OSP**	22.18 ± 0.88^cD^	21.31 ± 0.85^dC^	20.98 ± 0.62^dBC^	19.65 ± 0.64^dB^	18.70 ± 0.56^dA^
***b∗***	**Control**	16.29 ± 0.57^cBC^	15.89 ± 0.55^cB^	15.75 ± 0.55^dB^	15.35 ± 0.53^dAB^	14.05 ± 0.52^cA^
	**E129**	15.75 ± 0.55^bcC^	15.29 ± 0.53^cBC^	14.47 ± 0.50^cB^	14.11 ± 0.49^cAB^	13.78 ± 0.48^cA^
	**1-OSP**	14.97 ± 0.52^bD^	14.37 ± 0.50^bcCD^	13.15 ± 0.46^bcBC^	12.91 ± 0.45^bcB^	12.02 ± 0.45^bA^
	**2-OSP**	13.63 ± 0.47^abE^	13.02 ± 0.45^bD^	12.63 ± 0.44^bC^	11.93 ± 0.41^bB^	10.87 ± 0.38^aA^
	**4-OSP**	12.42 ± 0.43^aC^	11.58 ± 0.40^aB^	11.34 ± 0.39^aB^	11.06 ± 0.38^aAB^	10.74 ± 0.37^aA^
**Appearance**	**Control**	8.30 ±0.016^aD^	6.20 ± 0.080^aC^	5.06 ± 0.05^aB^	4.69 ± 0.06^aB^	3.54 ± 0.03^aA^
	**E129**	8.40 ± 0.012^abD^	6.78 ± 0.012^bC^	6.27 ± 0.15^bBC^	4.78 ± 0.04^abA^	4.58 ± 0.04^bA^
	**1-OSP**	8.45 ± 0.012^abE^	7.81 ± 0.016^cD^	6.32 ± 0.08^bC^	5.14 ± 0.05^bB^	4.74 ± 0.07^bcA^
	**2-OSP**	8.60 ± 0.016^bD^	7.83 ± 0.020^cC^	6.46 ± 0.09^bcBC^	6.16 ± 0.05^cB^	5.87 ± 0.05^cA^
	**4-OSP**	8.70 ± 0.012^bD^	8.12 ± 0.035^dC^	7.85 ± 0.10^cB^	6.61 ± 0.06^dAB^	6.20 ± 0.05^dA^
**Color**	**Control**	8.00 ± 0.016^aD^	7.10 ± 0.080^aC^	5.25 ± 0.07^aB^	5.04 ± 0.03^aB^	4.54 ± 0.04^aA^
	**E129**	8.15 ± 0.024^bD^	7.71 ± 0.060^bC^	6.40 ± 0.05^bBC^	6.03 ± 0.05^bB^	5.08 ± 0.01^bA^
	**1-OSP**	8.21 ± 0.008^bcD^	7.88 ± 0.11^bBC^	6.52 ± 0.01^bB^	6.44 ± 0.07^cB^	5.64 ± 0.06^cA^
	**2-OSP**	8.25 ± 0.008^bcD^	7.91 ± 0.06^bC^	6.81 ± 0.02^bcBC^	6.56 ± 0.05^cB^	5.87 ± 0.06^cA^
	**4-OSP**	8.40 ± 0.008^bcC^	8.27 ± 0.05^cBC^	7.05 ± 0.04^cB^	6.84 ± 0.07^cdA^	6.72 ± 0.01^dA^
**Odor**	**Control**	8.00 ± 0.016^aD^	7.20 ± 0.03^aC^	5.41 ± 0.01^aBC^	5.27 ± 0.01^aB^	4.12 ± 0.02^aA^
	**E129**	8.11 ± 0.012^aD^	7.31 ± 0.02^abC^	5.51 ± 0.03^aB^	5.31 ± 0.02^bB^	4.15 ± 0.03^aA^
	**1-OSP**	8.13 ± 0.012^aC^	7.80 ± 0.03^abcC^	7.06 ± 0.06^bB^	6.88 ± 0.03^cB^	5.61 ± 0.06^bA^
	**2-OSP**	8.20 ± 0.012^aC^	8.00 ± 0.06^bcC^	7.22 ± 0.05^bB^	7.11 ± 0.06^cB^	5.78 ± 0.04^bA^
**Overall acceptability**	**4-OSP**	8.32 ± 0.016^aC^	8.10 ± 0.05^cC^	7.85 ± 0.03^cBC^	7.51 ± 0.01^dB^	6.07 ± 0.01^cA^
	**Control**	8.00 ± 0.014^aD^	7.20 ± 0.01^aC^	6.44 ± 0.01^aBC^	5.82 ± 0.08^aB^	4.50 ± 0.05^aA^
	**E129**	8.12 ± 0.012^aE^	7.31 ± 0.08^aD^	6.73 ± 0.01^abC^	6.04 ± 0.05^abB^	4.78 ± 0.08^abA^
	**1-OSP**	8.54 ± 0.016^bD^	7.71 ± 0.05^bC^	7.54 ± 0.07^bC^	6.74 ± 0.07^bB^	5.27 ± 0.06^bA^
	**2-OSP**	8.60 ± 0.008^bD^	8.00 ± 0.02^cC^	7.73 ± 0.02^bcC^	6.94 ± 0.09^bcB^	5.49 ± 0.08^bA^
	**4-OSP**	8.75 ± 0.021^bD^	8.19 ± 0.01^Ccd^	7.95 ± 0.04^bcC^	7.11 ± 0.02^cB^	5.85 ± 0.01^bcA^

±, standard deviation (SD) of the three replicates; a–d: Mean values that are followed by a similar letter in the same column differ significantly (*P* < 0.05); A–E: Mean values that are not followed by a similar letter in the same line differ significantly (*P* < 0.05); S: Storage; T: Treatment; n.s: Not Significant; ∗*P* < 0.05; ∗∗*P* < 0.01 and ∗∗∗*P* < 0.001.

Table 3 illustrates an increase in PTC growth for all samples at different sampling times. From the beginning of the storage, meat treated with OSP extract depicted the lowest PTC. Remarkably, when OSP extract concentration increased the PTC growth in meat was significantly delayed (*P* < 0.05). After 14 days, 1-OSP, 2-OSP and 4-OSP meat-treated extracts exhibited PTC levels of 5.78, 5.51 and 5.05 log CFU/g, respectively. Intriguingly, the PTC in these meat samples did not reach the 6.7 log CFU/g threshold [62] when OSP extracts were used. Thus, these extracts showed an extended meat shelf life, lasting up to 14 days at 4 °C.

Hence, correlation of the impact of OSP extracts with the dosage and duration of storage occurred. Our findings align with the outcomes reported earlier by Palmeri et al. [64] in the context of sliced beef, which demonstrated the efficacy of prickly pear extract (PPE) in significantly reducing bacterial growth during storage at 4 °C. Additionally, Kharrat et al. [65] reported that red prickly pear (*Opuntia stricta*) at 2.5 % inhibited bacterial growth in salami stored at 4 °C over 30 days. Likewise, incorporating OSP enhances the microbiological stability of meat and meat products, likely attributed to the abundance of flavonoids, betalains, and phenolic molecules.

The concentration-dependent reduction in APC levels highlights the dose-response relationship between OSP extract concentration and its antibacterial activity. The use of OSP extract in meat products may be an effective means to increase shelf life and guarantee food safety, satisfying consumer desire for natural and clean-label food preservation techniques.

#### 3.4.2 Physiochemical analysis

- Evaluation of chemical oxidation products

During chilling, a statistically significant increase in carbonyl content was observed (Table 3). Control meat samples and those treated with E129 exhibited a higher level of protein carbonylation compared to treated samples with 1-4-OSP all over the storage period (*P* < 0.05). Moreover, OSP extracts proved to show a beneficial effect in delaying carbonylation. An increase in the concentration of OSP extract showed a decrease in the perceived level of protein carbonylation. In fact, the antioxidant properties of the OSP extract contributed to delaying protein oxidation, thus extending the red color of meats. The exceptional efficacy of OSP extract could be ascribed to its high betalain content, exhibiting metal-chelating activity and thereby providing superior protection against protein oxidation [66].

Regarding lipid oxidation, Table 3 illustrates a significant (*P* < 0.05) increase in peroxide values across all samples during chilled storage, with the control and E129-treated samples exhibiting the highest PV. The meat sample treated with 4-OSP extract displayed the lowest peroxide value (6.14 meq O_2_/kg), followed by the 2-OSP extract (6.64 meq O_2_/kg) and the 1-OSP extract (7.53 meq O_2_/kg) reaching the 14th day of storage. Furthermore, a significant increase (*P* < 0.05) in conjugated dienes was observed for the initial 3 days in all meat samples (Table 3). This phenomenon was attributed to the faster formation of conjugated dienes compared to hydroperoxides decomposition. The generation of secondary lipid oxidation products, specifically aldehydes and ketones could explain the subsequent decrease in conjugated dienes [67]. Furthermore, a decline in conjugated dienes was noted in meat samples treated with 1-OSP, 2-OSP, and 4-OSP extracts during storage. This suggests that OSP extracts may reduce lipid oxidation by delaying the formation of conjugated dienes in meat products. Additionally, TBARS values exhibited a significant increase (*P* < 0.05) in all treated meat samples during storage (Table 3). Higher TBARS values were observed for the control sample (2.13 mg MDA eq/kg of meat) and meat treated with E129 (2.05 mg MDA eq/kg of meat), exceeding the specified limit of TBARS (2 mg MDA eq/Kg of meat reaching the 10th day of storage [24]. The TBARS levels for 1-OSP, 2-OSP, and 4-OSP extracts-treated meat samples were 1.41, 1.29 and 1.14 mg MDA eq/kg of meat, respectively. Notably, an increase in OSP extract concentration led to a decrease in TBARS levels showing the dose-dependent antioxidant effect of OSP extracts in mitigating lipid oxidation in meat products. The effect of delaying the lipid and protein oxidation of meat would be due to the antioxidant activity of OSP extract.

- Meat color analysis

As per the data presented in Table 3, *L*∗, *a*∗, and *b*∗ exhibited a significant (*P* < 0.05) decrease with the progression of storage time. The L∗ value of control meat samples was higher (*P* < 0.05) than those treated with E129, 1-OSP, 2-OSP, and 4-OSP extracts. In fact, as reported by Hughes et al. [68], a higher total pigment content suggested increased absorbance and reduced total reflectance, resulting in a darker sample and decreased lightness. In terms of *a*∗ values, the incorporation of OSP extract significantly (*P* < 0.05) intensified the red color of meat samples compared to other samples. For instance, at the beginning of storage meat samples treated with 1-OSP, 2-OSP, and 4-OSP extracts presented a value of *a∗* equal to 17.68, 18.49 and 22.18, respectively. Even after 14 days of storage, these treated meat samples 1-OSP, 2-OSP, and 4-OSP extracts maintained enhanced red coloration, with respective *a*∗ values of 11.92, 14.51 and 18.70. These results underscore the highly effective betalain content in imparting and preserving the desired red color in meat. Similar trends were suggested by Kharrat et al. [65], who mentioned that prickly pear extract incorporated into salami products displayed a change in color depending (1 or 2.5 %) on the extract concentration added.

A decline was observed for *b*∗ values of non-treated meat samples, reaching 15.05 on the 14th day of storage whereas treated meat samples exhibited a slight decrease in *b*∗. This observation aligns with a study by Palmeri et al. [64], which reported that prickly pear extract (PPE) addition to sliced beef meat showed a similar trend in *a*∗ and b∗ values. The remarkable changes in *L∗*, *a*∗ and *b∗* indices during storage could be attributed to lipid oxidation, where primary lipid oxidation products such as hydroperoxides and other reactive oxygen species oxidize ferrous iron (Fe^2+^) from oxymyoglobin (OxyMb) to ferric iron (Fe^3+^) in metmyoglobin (MetMb) [69].

#### 3.4.3 Sensory assessment

The sensory quality of meat, characterized by its desirable odor and color development, plays a pivotal role in overall consumer acceptance. The incorporation of OSP extract significantly enhanced the acceptability of meat, with color attributes serving as crucial indicators for consumer preference or rejection, as noted in studies such as Martins et al. [70]. The appearance, odor, color, and overall acceptability scores of beef meat enriched with E129 and OSP extract surpassed those of control samples (Table 3). This improvement is attributed to the betalains pigments present in OSP extract, imparting a naturally vibrant pink hue in the treated meat samples. Our findings align with Kharrat et al. [65] study, who reported that the scores for color and odor of salami formulated with prickly pear extracts influenced the panellist acceptability—increased with the extract concentration. These authors revealed that the salami formulated with prickly pear extract at 2.5 % presented a higher score than the control. As depicted in Table 3, all sensory attributes exhibited a significant reduction (*P* < 0.05) during refrigerated storage, potentially attributable to increased oxidation and microbial growth over time. These results prove that the addition of OSP extract not only improves the visual appeal of meat products but also positively impacts other sensory attributes like odor and overall acceptability.

### 3.5 Chemometric analysis

#### 3.5.1 Principal component analysis

**Fig. 4:**
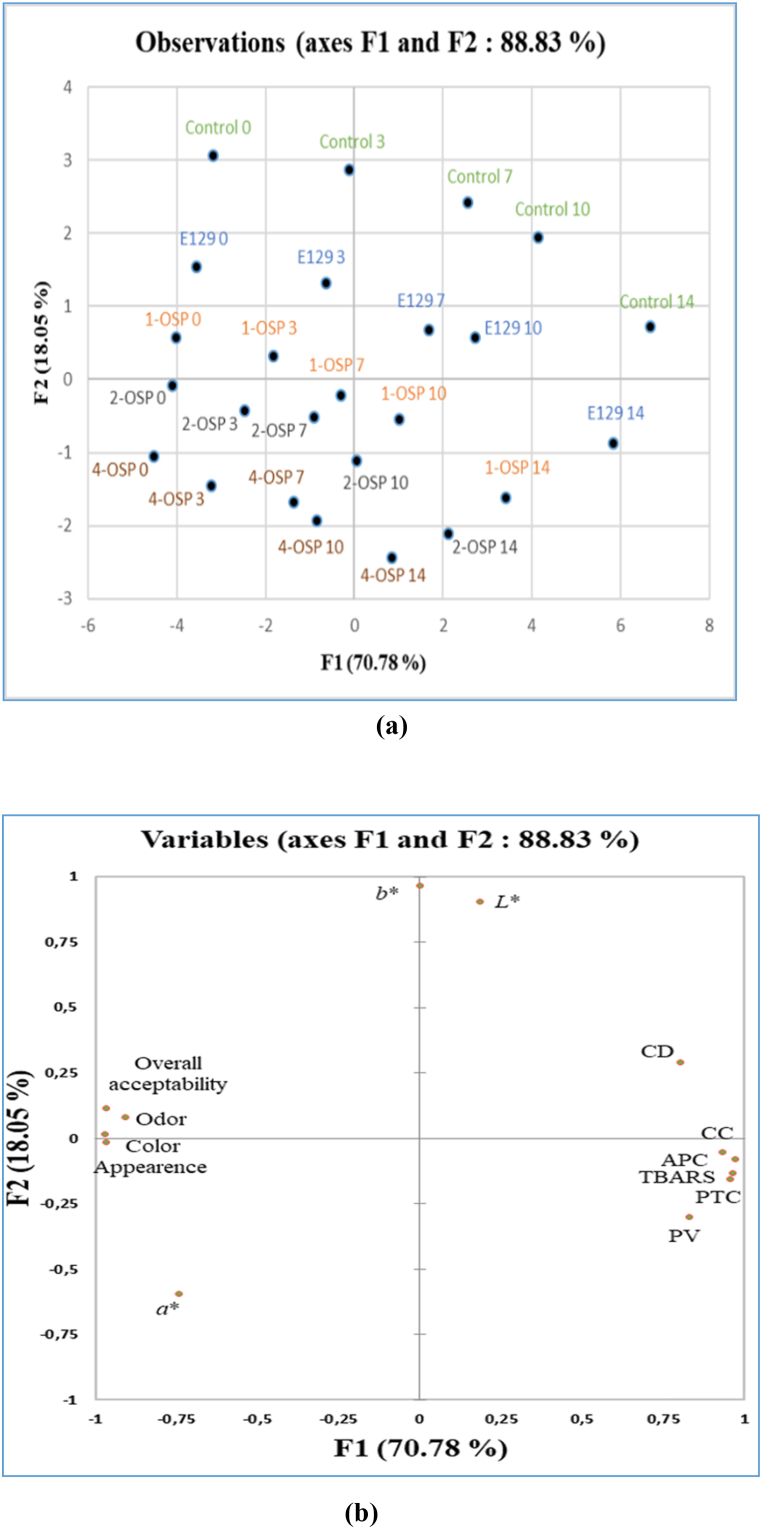
Loading plots of the two principal components (F1, F2) based on all samples (**a)** and on physicochemical, microbial growth, instrumental color, and sensorial property values of raw meat samples over 14 days of storage (**b**).

The PCA technique was utilized to gain an overall understanding of the distinctions and similarities among five samples and their influence on minced beef meat's instrumental color, lipid/protein oxidation, microbial growth, and sensory parameters across five-time points. A biplot of the PCA loadings for two components (F1 and F2) is shown in Fig. 4a and b. F1 effectively accounted for 70.78 % of the total variation of the dataset, while F2 contributed an additional 18.05 % to the study's variation. The interpretation of measurements and principal components (PCs) relies on the correlations between each parameter and each PC. Parameters that are closely aligned are positively correlated, those separated by 180° are negatively correlated, and if they are 90° apart, they are considered independent [71]. Accordingly, the lipid and protein oxidation parameters were negatively correlated with sensory attributes. In addition, according to Fig. 4b, control samples and E129 were placed on the upper side of the biplot where *L*∗ and *b*∗ were placed, while 2-OSP and 4-OSP samples were placed in the bottom where sensory attributes, as well as *a*∗, were placed.

#### 3.5.2 Heat maps

**Fig. 5:**
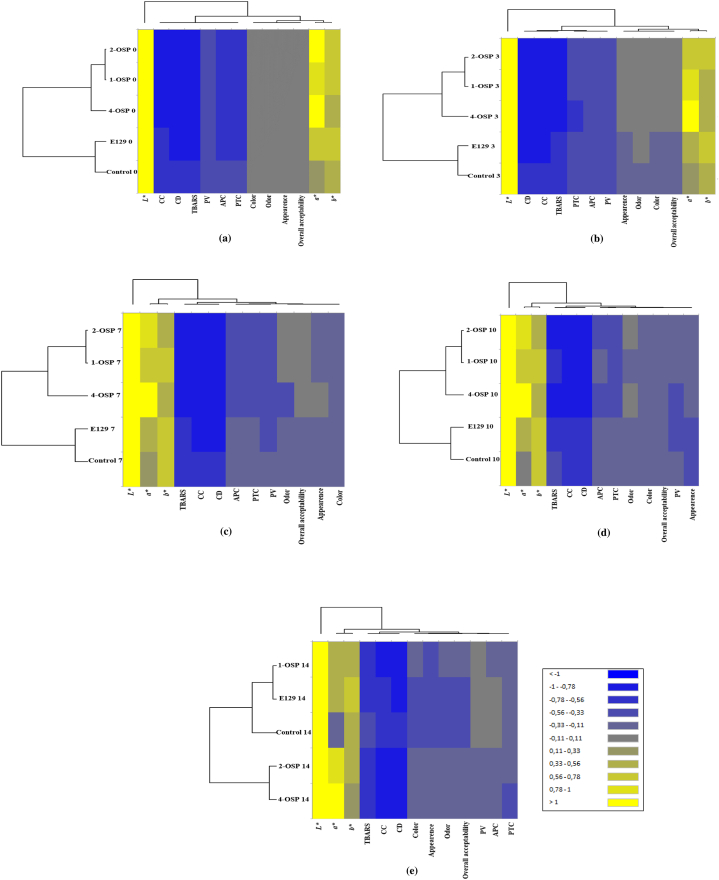
Heat maps of physicochemical, microbial growth, instrumental color, and sensorial property values of raw meat samples on (**a**) day 0; (**b)** day 3; (**c)** day 7; (**d**) day 10; and (**e)** day 14 of storage.

Fig. 5(a–e) depict two clusters, one of them regroups 1-OSP, 2-OSP, and 4-OSP indicating a similarity between those clusters. Protein oxidation and microbial growth are strongly associated in meat products from day 0 of storage. The oxidation products can impact microbial growth. The spoilage of raw beef is primarily caused by undesired microbial development during storage, with different spoilage-related microbial populations developing based on storage conditions [72]. *a*∗ and *b*∗ color indices, especially on day 3, are correlated with sensory attributes of beef meat. In fact, changes in *a*∗ value can be observed by panelists as indicated by Mancini et al. [73].

On days 7, 10 and 14, there is a noted correlation between sensory properties and microbial growth. Mojaddar Langroodi et al. [74] also mentioned an interconnection between sensory properties and microbiological investigations. Meat samples exhibited unsatisfactory sensory properties, including off-aroma, off-colors, and an undesirable visual appearance, resulting in lower scores due to the high presence of microorganisms and lipid oxidation [75]. Moreover, sensory attributes were correlated with elevated microbial counts and parameters associated with primary and secondary lipid oxidation, as well as protein oxidation. These interconnected characteristics collectively influence meat quality. Also as mentioned in the heat maps at all storage periods, a correlation between CC, CD, and TBARS values was observed. A significant and positive relationship between CC and TBARS was supported by Zhao et al. [76] corroborating this correlation. These authors observed higher TBARS levels at elevated protein oxidation levels, resulting in strong association of lipid and protein oxidation [76]. Thus, these techniques offer valuable insights into the changes in meat quality over time, providing a comprehensive understanding of the factors influencing meat freshness and spoilage during storage. It is evident that chemometric tools can be widely employed in the assessment of meat quality based on its oxidative stability and color characteristics during storage [77,78].

## 4 Conclusion

This study demonstrates the potential application of betalains from OSP as a natural colorant in the meat industry. By using UPLC-MS/MS, seven metabolites were identified as six betacyanins and an arginine-betaxanthin (as betaxanthin). By skillfully using *in silico* and software tools, we demonstrated that the cyclo-Dopa-5-O-β-glucoside and arginine-betaxanthin could inhibit *L. monocytogenes* replication and transcription functions by targeting the DHFR, suggesting their potential as a beneficial additive in meat preservation. Practically, the use of OSP extract at 0.012 % in raw minced beef meat may be an effective means to increase its shelf life and guarantee food safety, satisfying consumer desire for natural and clean-label food preservation techniques. The corresponding concentration (OSP at 0.012 %) limited microbial deterioration, delayed chemical oxidation, and enhanced instrumental color and sensory traits. Additionally, at each storage time, PCA and heat maps revealed different correlations between lipid/protein oxidation and microbial counts, instrumental color measurements and sensory features. This investigation underscores OSP betalains as a natural and efficient substitute for enhancing meat quality and safety, fulfilling both consumer expectations and industry sustainability objectives. Furthermore, assessing the potential use of OSP extract in meat products after cooking and processing is essential to determine their stability and effectiveness in retaining visual appeal post-heat treatment. Cooking methods can alter the appearance of meat, and it is crucial to ensure that OSP extract can withstand these conditions while maintaining its color vibrancy. Understanding how the extract performs after processing will provide insights into its application, reinforcing its role in improving both the quality of meat products, while meeting consumer demands for natural ingredients and cleaner labels.

## Funding

This work is supported through Tunisian-Algerian Project 2020 for cooperation in the field of research and innovation (Order number: C120211040000002161900015).

## CRediT authorship contribution statement

**Moufida Chaari:** Writing – original draft, Investigation, Conceptualization. **Sarra Akermi:** Software, Methodology. **Khaoula Elhadef:** Resources, Formal analysis. **Monia Ennouri:** Methodology, Data curation. **Lobna Jlaiel:** Formal analysis, Data curation. **Mohamed Ali Mosrati:** Investigation, Data curation. **Lotfi Mellouli:** Visualization. **Walid Elfalleh:** Software, Resources, Funding acquisition, Data curation. **Theodoros Varzakas:** Visualization, Project administration. **Slim Smaoui:** Writing – review & editing, Writing – original draft, Project administration, Conceptualization.

## Declaration

The experimental scheme involving sensory evaluation does not need ethical approval. In the course of the implementation of this study, no human body, animal violation, or morality was involved. Additionally, participants were not harmed or affected in any way by being included in this study, nor were any personal or confidential data disclosed.

In this study, no physical or psychological harm was caused to participants. There was no violation of personal rights, as no personal or confidential information was disclosed.

Participation in the sensory evaluation was entirely voluntary, and participants were fully informed of the nature of the study beforehand. Given these factors and the non-invasive, non-harmful nature of the sensory evaluation, ethical approval was not deemed necessary. However, we assure that participants were treated in accordance with best practices, and written informed consent was obtained from all participants prior to their involvement in the study.

## Data availability statement

Data will be made available on request.

## Declaration of competing interest

The authors declare that they have no known competing financial interests or personal relationships that could have appeared to influence the work reported in this paper.
